# Oodles of opportunities: the Journal of Cachexia, Sarcopenia and Muscle in 2017

**DOI:** 10.1002/jcsm.12247

**Published:** 2017-10-27

**Authors:** Stephan von Haehling, Nicole Ebner, Stefan D. Anker

**Affiliations:** ^1^ Department of Cardiology and Pneumology University of Göttingen Medical Center Göttingen Germany; ^2^ Division of Cardiology and Metabolism—Heart Failure, Cachexia & Sarcopenia, Department of Cardiology (CVK); and Berlin‐Brandenburg Center for Regenerative Therapies (BCRT); Deutsches Zentrum für Herz‐Kreislauf‐Forschung (DZHK) Berlin Charité Universitätsmedizin Berlin Berlin Germany

The good news first, the *Journal of Cachexia, Sarcopenia and Muscle* (JCSM) has not only maintained its impact factor, but it has even increased it again, now reaching 9.697 as has been published by Thomson Scientific a few weeks ago. To obtain a grip of the impact factor, it has to be acknowledged that it requires calculation of cites to items published in 2014 and 2015 divided by the number of items published in 2014 and 2015. In numbers, we reached a total of 368 cites in 2014 and 272 cites in 2015, summing up to 640 cites in total. This may not seem a lot, but considering that we only published 66 items deemed countable (editorial comments and letters‐to‐the‐editor are not counted), the final impact factor reached 9.697, implying that each of our papers are cited almost 10 times over the course of 2 years. This places JCSM as the number 8 ranked journal among all journals in the category ‘Medicine, General and Internal’ (Table 1) and as number 2 ranking publication among all nutrition journals, among which, however, JCSM is still not officially listed by Thomson Scientific (Table 2).

As we have done before and are not getting tired of, we herewith express our gratitude to all authors, reviewers, and editorial board members for their great efforts to produce JCSM at good quality, and we greatly appreciate and value also the interest and support of all those who enjoy reading JCSM and cite the papers published there. Of course, the Journal would not be what it is without our editorial office team Monika Diek and Corinna Denecke, and we would also like to express our thanks for their professional support!

**Table 1:** Top 10 journals in the field ‘medicine: General & Internal’

		**Impact**	**Items**	Issues
	**Journal name**	**factor**	**published**	per
		**2017**	**2015 and 2016**	Year
1	New England Journal of Medicine	72.406	670	52
2	Lancet	47.831	646	52
3	Journal of the American Medical Association (JAMA)	44.405	410	48
4	British Medical Journal	20.785	446	52
5	Annals of Internal Medicine	17.135	150	24
6	JAMA Internal Medicine	16.538	275	12
7	PLOS Medicine	11.862	286	12
8	Journal of Cachexia, Sarcopenia and Muscle	9.697	88	4
9	BMC Medicine	7.901	418	
10	Journal of Internal Medicine	7.598	194	12

**Table 2:** Top 10 journals in the field ‘Nutrition & Dietetics’, where the Journal of Cachexia, Sarcopenia and Muscle is officially not listed

		**Impact**	**Items**	Issues
	**Journal name**	**factor**	**published**	per
		**2017**	**2015 and 2016**	Year
1	Progress in Lipid Research	10.583	69	4
2	Annuals Review of Nutrition	9.054	44	1
3	American Journal of Clinical Nutrition	6.926	649	12
4	Critical Reviews in Food Science and Nutrition	6.077	149	12
5	International Journal of Obesity	5.487	502	12
6	Nutrition Reviews	5.291	132	12
7	Advances in Nutrition	5.233	177	6
8	Nutrition Research Reviews	4.844	29	2
9	Clinical Nutrition	4.548	375	6
10	Food Chemistry	4.529	3547	24

This year is special to JCSM for several reasons. One is the publication of additional issues this year, the other is the inauguration of our two daughter journals. Indeed, JCSM appears to have sparked more scientific interest in the field of body wasting, cachexia, and sarcopenia, and thus the number of submissions to the main journal remains on the increase. With a 73% rejection rate, we are well aware of the fact that we have to decline publication of many good papers, simply for lack of space. However, we do hope that we are able to give some of these a home in our daughter journals—*JCSM Rapid Communications* and *JCSM Clinical Reports*. The latter is online already since December 2016, and a good number of original research papers has been published since. At the time of writing this editorial in August 2017, the main journal, JCSM, has received already 187 submissions in 2017 alone, proving a steady increase in submissions: last year, this number was ‘only’ 158. Given these higher numbers of submissions, in 2017 we will move to six issues per year to allow publication of more accepted papers.

We are working hard to provide a timely peer review, which is not always easy, as it is difficult to find appropriate reviewers at times. Articles that are available for the longest time are—not surprisingly—those that have been cited most (Table 3). Our ‘facts and numbers’ editorials remain popular (Tables 4 and 5), and we invite our readers to submit their work or to suggest topics for ‘facts and numbers’ editorials that are relevant to our readers (Table 6).

**Table 3:** Top 10 of best cited articles since first publication of the Journal of Cachexia, Sarcopenia and Muscle

	**First Author**	**Title**	**Type**	**Year**	**Times Cited**	**Reference**
1	von Haehling	Cachexia as a major underestimated and unmet medical need: facts and numbers	Editorial	2010	203	1
2	von Haehling	An overview of sarcopenia: facts and numbers on prevalence and clinical impact	Editorial	2010	125	2
3	Dalton	The selective androgen receptor modulator GTx‐024 (enobosarm) improves lean body mass and physical function in healthy elderly men and postmenopausal women: results of a double‐blind, placebo‐controlled phase II trial	Original article	2011	111	3
4	Morley	From Sarcopenia to frailty: a road less travelled	Editorial	2014	94	4
5	Lenk	Skeletal muscle wasting in cachexia and sarcopenia: molecular pathophysiology and impact of exercise training	Review	2010	90	5
6	Fanzani	Molecular and cellular mechanisms of skeletal muscle atrophy: an update	Review	2012	78	6
7	Elkina	The role of myostatin in muscle wasting: an overview.	Review	2011	75	7
8	Cesari	Biomarkers of sarcopenia in clinical trials‐recommendations from the International Working Group on Sarcopenia	Original article	2012	72	8
9	Mak	Wasting in chronic kidney disease	Review	2011	67	9
10	von Haehling	From muscle wasting to sarcopenia and myopenia: update 2012	Editorial	2012	63	10

**Table 4:** Top 20 of best cited articles published 2014 in the Journal of Cachexia, Sarcopenia and Muscle

	First Author	Title	Type	Times cited	Reference
1	Wakabayashi	Rehabilitation nutrition for sarcopenia with disability: a combination of both rehabilitation and nutrition care management	Review	79	11
2	von Haehling	Prevalence, incidence, and clinical impact of cachexia: facts and numbers—update 2014	Editorial	75	12
3	Morley	Prevalence, incidence, and clinical impact of sarcopenia: facts, numbers, and epidemiology—update 2014	Editorial	69	13
4	Morley	From sarcopenia to frailty: a road less travelled	Editorial	59	4
5	Ormsbee	Osteosarcopenic obesity: the role of bone, muscle, and fat on health	Review	39	14
6	Heymsfield	Assessing skeletal muscle mass: historical overview and state of the art	Review	37	15
7	Morley	Are we closer to having drugs to treat muscle wasting disease?	Editorial	36	16
8	Anker	Muscle wasting disease: a proposal for a new disease classification	Editorial	27	17
9	Ebner	Highlights from the 7th Cachexia Conference: muscle wasting pathophysiological detection and novel treatment strategies	Meeting Report	26	18
10	Pietra	Anamorelin HCl (ONO‐7643), a novel ghrelin receptor agonist, for the treatment of cancer anorexia‐cachexia syndrome: preclinical profile	Original Article	26	19
11	Fragala	Biomarkers of muscle quality: N‐terminal propeptide of type III procollagen and C‐terminal agrin fragment responses to resistance exercise training in older adults	Original Article	25	20
12	Palus	Muscle wasting: an overview of recent developments in basic research	Review	20	21
13	Josiak	Skeletal myopathy in patients with chronic heart failure: significance of anabolic‐androgenic hormones	Review	20	22
14	Toledo	Formoterol in the treatment of experimental cancer cachexia: effects on heart function	Original Article	19	23
15	Alchin	Sarcopenia: describing rather than defining a condition	Review	17	24
16	Argiles	Cachexia: a problem of energetic inefficiency	Review	16	25
17	Rhee	Resistance exercise: an effective strategy to reverse muscle wasting in hemodialysis patients?	Editorial	15	26
18	Khawaja	Ventricular assist device implantation improves skeletal muscle function, oxidative capacity, and growth hormone/insulin‐like growth factor‐1 axis signalling in patients with advanced heart failure	Original Article	15	27
19	Mirza	Attenuation of muscle wasting in murine C2C12 myotubes by epigallocatechin‐3‐gallate	Original Article	15	28
20	Kirkman	Anabolic exercise in haemodialysis patients: a randomized controlled pilot study	Original Article	13	29

**Table 5:** Top 20 of best cited articles published 2015 in the Journal of Cachexia, Sarcopenia and Muscle

	First Author	Title	Type	Times cited	Reference
1	Calvani	Biomarkers for physical frailty and sarcopenia: state of the science and future developments	Review	37	30
2	Bowen	Skeletal muscle wasting in cachexia and sarcopenia: molecular pathophysiology and impact of exercise training	Review	36	31
3	Ezeoke	Pathophysiology of anorexia in the cancer cachexia syndrome	Review	28	32
4	Fearon	Request for regulatory guidance for cancer cachexia intervention trials	Editorial	24	33
5	Chen	Ghrelin prevents tumour‐induced and cisplatin‐induced muscle wasting: characterization of multiple mechanisms involved	Original Article	17	34
6	Manger	Skeletal muscle alterations in chronic heart failure: differential effects on quadriceps and diaphragm	Original Article	17	35
7	Grande	Exercise for cancer cachexia in adults: Executive summary of a Cochrane Collaboration systematic review	Review	16	36
8	Sasso	A framework for prescription in exercise‐oncology research	Editorial	14	37
9	Cvan Trobec	Influence of cancer cachexia on drug liver metabolism and renal elimination in rats	Original Article	13	38
10	Dupuy	Searching for a relevant definition of sarcopenia: results from the cross‐sectional EPIDOS study	Original Article	13	39
11	Morley	Rapid screening for sarcopenia	Editorial	12	40
12	Stephens	Evaluating potential biomarkers of cachexia and survival in skeletal muscle of upper gastrointestinal cancer patients	Original Article	11	41
13	Faber	Improved body weight and performance status and reduced serum PGE2 levels after nutritional intervention with a specific medical food in newly diagnosed patients with esophageal cancer or adenocarcinoma of the gastro‐esophageal junction	Original Article	10	42
14	Drescher	Loss of muscle mass: current developments in cachexia and sarcopenia focused on biomarkers and treatment	Review	8	43
15	Wakabayashi	Skeletal muscle mass is associated with severe dysphagia in cancer patients	Original Article	8	44
16	Dev	Hypermetabolism and symptom burden in advanced cancer patients evaluated in a cachexia clinic	Original Article	6	45
17	Cooper	Understanding and managing cancer‐related weight loss and anorexia: insights from a systematic review of qualitative research	Review	6	46
18	Marino	Activin‐βC modulates cachexia by repressing the ubiquitin‐proteasome and autophagic degradation pathways	Original Article	6	47
19	Haruta	One‐year intranasal application of growth hormone releasing peptide‐2 improves body weight and hypoglycemia in a severely emaciated anorexia nervosa patient	Original Article	5	48
20	van Norren	Behavioural changes are a major contributing factor in the reduction of sarcopenia in caloric‐restricted ageing mice	Original Article	5	49

**Table 6:** Top 20 of best cited articles published 2016 in the Journal of Cachexia, Sarcopenia and Muscle

	First Author	Title	Type	Times cited	Reference
1	Malmstrom	SARC‐F: a symptom score to predict persons with sarcopenia at risk for poor functional outcomes	Original Article	33	50
2	Tyrovolas	Factors associated with skeletal muscle mass, sarcopenia, and sarcopenic obesity in older adults: a multi‐continent study	Original Article	12	51
3	Sakuma	p62/SQSTM1 but not LC3 is accumulated in sarcopenic muscle of mice	Original Article	9	52
4	Loncar	Cardiac cachexia: hic et nunc	Review	8	53
5	Go	Prognostic impact of sarcopenia in patients with diffuse large B‐cell lymphoma treated with rituximab plus cyclophosphamide, doxorubicin, vincristine, and prednisone	Original Article	6	54
6	de Lima	Doxorubicin caused severe hyperglycaemia and insulin resistance, mediated by inhibition in AMPK signalling in skeletal muscle	Original Article	6	55
7	Lodka	Muscle RING‐fingers 2 and 3 maintain striated‐muscle structure and function	Original Article	5	56
8	Lewis	Increased expression of H19/miR‐675 is associated with a low fat‐free mass index in patients with COPD	Original Article	5	57
9	Montano‐Loza	Sarcopenic obesity and myosteatosis are associated with higher mortality in patients with cirrhosis	Original Article	4	58
10	Barbosa‐Silva	Prevalence of sarcopenia among community‐dwelling elderly of a medium‐sized South American city: results of the COMO VAI? study	Original Article	4	59
11	Penna	Effect of the specific proteasome inhibitor bortezomib on cancer‐related muscle wasting	Original Article	4	60
12	Vries	Patient‐centred physical therapy is (cost‐) effective in increasing physical activity and reducing frailty in older adults with mobility problems: a randomized controlled trial with 6 months follow‐up	Original Article	4	61
13	Batista	Cachexia‐associated adipose tissue morphological rearrangement in gastrointestinal cancer patients	Original Article	3	62
14	Giron	Conversion of leucine to β‐hydroxy‐β‐methylbutyrate by α‐keto isocaproate dioxygenase is required for a potent stimulation of protein synthesis in L6 rat myotubes	Original Article	3	63
15	Lainscak	ACT‐ONE‐ACTION at last on cancer cachexia by adapting a novel action beta‐blocker	Editorial	3	64
16	Berger	Dysfunction of respiratory muscles in critically ill patients on the intensive care unit	Review	3	65
17	Musolino	Megestrol acetate improves cardiac function in a model of cancer cachexia‐induced cardiomyopathy by autophagic modulation	Original Article	3	66
18	Neves	White adipose tissue cells and the progression of cachexia: inflammatory pathways	Original Article	2	67
19	Polge	UBE2B is implicated in myofibrillar protein loss in catabolic C2C12 myotubes	Original Article	2	68
20	Pinto	Impact of creatine supplementation in combination with resistance training on lean mass in the elderly	Original Article	2	69

Finally, we would like to draw attention to the upcoming Cachexia Conference to be held between December 8–10, 2017 in Rome, Italy. The conference became an annual meeting already 2 years ago, and it is a source of stimulating ideas and exchange between clinicians and researchers in the field of cachexia and wasting. Data on the final program and more information is to be found at http://society‐scwd.org.
